# The serum of COVID-19 asymptomatic patients up-regulates proteins related to endothelial dysfunction and viral response in circulating angiogenic cells ex-vivo

**DOI:** 10.1186/s10020-022-00465-w

**Published:** 2022-04-09

**Authors:** Lucía Beltrán-Camacho, Sara Eslava-Alcón, Marta Rojas-Torres, Daniel Sánchez-Morillo, Mª Pilar Martinez-Nicolás, Victoria Martín-Bermejo, Inés García de la Torre, Esther Berrocoso, Juan Antonio Moreno, Rafael Moreno-Luna, Mª Carmen Durán-Ruiz

**Affiliations:** 1 Biomedicine, Biotechnology and Public Health Department, University of Cadiz, 11002 Cadiz, Spain; 2 Biomedical Research and Innovation Institute of Cadiz (INiBICA), 11002 Cadiz, Spain; 3 Automation Engineering, Electronics and Computer Architecture and Networks Department, University of Cadiz, 11009 Cadiz, Spain; 4 Occupational Health Service, National Paraplegic Hospital, SESCAM, 45071 Toledo, Spain; 5 Department of Nursing, National Paraplegic Hospital, SESCAM, 45004 Toledo, Spain; 6 Physiotherapy Service, National Paraplegic Hospital, SESCAM, 45071 Toledo, Spain; 7 Psychology Department, University of Cádiz, 11510 Puerto Real, Spain; 8 Biomedical Research Networking Center for Mental Health Network (CIBERSAM), Institute of Health Carlos III, 28029 Madrid, Spain; 9 Maimonides Biomedical Research Institute of Cordoba (IMIBIC), UGC Nephrology, Reina Sofía University Hospital, 14004 Cordoba, Spain; 10 Cell Biology, Physiology and Immunology Department, Agrifood Campus of International Excellence (ceiA3), University of Cordoba, 14014 Córdoba, Spain; 11 Laboratory of Neuroinflammation, National Paraplegic Hospital, SESCAM, 45071 Toledo, Spain; 12 Biomedicine, Biotechnology and Public Health Department, Science Faculty, Cádiz University, Torre Sur. Avda. República Saharaui S/N, Polígono Río San Pedro, Puerto Real, 11519 Cádiz, Spain

**Keywords:** COVID-19, SARS-CoV-2, Endothelial progenitor cells, Circulating angiogenic cells, Proteomics, Endothelial dysfunction, Asymptomatic

## Abstract

**Background:**

Severe acute respiratory syndrome coronavirus 2 (SARS-CoV-2) has already caused 6 million deaths worldwide. While asymptomatic individuals are responsible of many potential transmissions, the difficulty to identify and isolate them at the high peak of infection constitutes still a real challenge. Moreover, SARS-CoV-2 provokes severe vascular damage and thromboembolic events in critical COVID-19 patients, deriving in many related deaths and long-hauler symptoms. Understanding how these processes are triggered as well as the potential long-term sequelae, even in asymptomatic individuals, becomes essential.

**Methods:**

We have evaluated, by application of a proteomics-based quantitative approach, the effect of serum from COVID-19 asymptomatic individuals over circulating angiogenic cells (CACs)*.* Healthy CACs were incubated ex-vivo with the serum of either COVID-19 negative (PCR −/IgG −, n:8) or COVID-19 positive asymptomatic donors, at different infective stages: PCR +/IgG − (n:8) and PCR −/IgG + (n:8). Also, a label free quantitative approach was applied to identify and quantify protein differences between these serums. Finally, machine learning algorithms were applied to validate the differential protein patterns in CACs.

**Results:**

Our results confirmed that SARS-CoV-2 promotes changes at the protein level in the serum of infected asymptomatic individuals, mainly correlated with altered coagulation and inflammatory processes (Fibrinogen, Von Willebrand Factor, Thrombospondin-1). At the cellular level, proteins like ICAM-1, TLR2 or Ezrin/Radixin were only up-regulated in CACs treated with the serum of asymptomatic patients at the highest peak of infection (PCR + /IgG −), but not with the serum of PCR −/IgG + individuals. Several proteins stood out as significantly discriminating markers in CACs in response to PCR or IgG + serums. Many of these proteins particiArticle title: Kindly check and confirm the edit made in the article title.pate in the initial endothelial response against the virus.

**Conclusions:**

The ex vivo incubation of CACs with the serum of asymptomatic COVID-19 donors at different stages of infection promoted protein changes representative of the endothelial dysfunction and inflammatory response after viral infection, together with activation of the coagulation process. The current approach constitutes an optimal model to study the response of vascular cells to SARS-CoV-2 infection, and an alternative platform to test potential inhibitors targeting either the virus entry pathway or the immune responses following SARS-CoV-2 infection.

**Supplementary Information:**

The online version contains supplementary material available at 10.1186/s10020-022-00465-w.

## Background

Severe acute respiratory syndrome coronavirus 2 (SARS-CoV-2) is the pathogen responsible of the coronavirus disease 2019 (COVID-19), declared as a global pandemic on March 11, 2020, by the World Health Organization. SARS-CoV-2 was identified for the first time in hospitalized patients with pneumonia in Wuhan (China) in December 2019, as an RNA virus of coronaviruses family (Zhu et al. 2020). Up to date (March 3, 2022), COVID-19 has provoked 6 million deaths worldwide (www.covid19.who.int), significantly affecting public health, the economics and society (Shipton et al. 2021; Bambra et al. 2020). Asymptomatic COVID-19 cases are responsible for many transmissions, which constitutes a real challenge to control the pandemic (Kronbichler et al. 2020). Approximately half of SARS-CoV-2 positive individuals are symptomatic at the time of testing, as determined by reverse transcriptase-polymerase chain reaction (RT-PCR) (Alene et al. 2021; Ra et al. 2021). This makes their detection quite difficult, since most of these individuals don’t seek testing and/or medical assistance and continue with their daily routine, contributing to rapid spread of COVID-19 (Gao et al. 2021). The identification of alternative markers (apart from physical symptoms or qPCR analysis) could significantly contribute to detect all potential SARS-CoV-2 infected individuals. Besides, little is known about the potential sequelae of SARS-Cov-2 over asymptomatic patients, and also how these initially “mild” infected people might become long-haulers at the long term (Huang et al. 2021).

Manifestations of COVID-19 are mostly respiratory; however, COVID-19 can also negatively affect extra-pulmonary systems (Snell 2021), including the heart and systemic vasculature (Klok et al. 2020; Marone and Rinaldi 2020; Huang et al. 2020). Indeed, SARS-CoV-2 infection has been linked to cardiovascular alterations (arrhythmias, ischemic heart disease or cardiomyopathies), mainly associated to coagulation abnormalities and endothelial damage, leading to thrombosis (Alvarado-Moreno et al. 2021; Thachil et al. 2020). COVID-19 enhances endothelial dysfunction, which not only involves oxidative stress, dysregulation of vascular tone or inflammatory response from the vascular wall (Jin et al. 2020), but also promotes the mobilization and recruitment of endothelial progenitor cells (EPCs) (Alvarado-Moreno et al. 2021; Mancuso et al. 2020), key cells involved in vascular repair (Zhang et al. 2014). Remarkably, the levels of circulating EPCs are significantly increased in the blood of COVID-19 patients compared with healthy controls (Mancuso et al. 2020; Guervilly et al. 2020), even three months after SARS-CoV-2 infection (Poyatos et al. 2021).

EPCs were first isolated from peripheral blood by Asahara et al., being defined as CD34 + cells that could differentiate in vitro to endothelial cells (ECs) (Asahara et al. 1997). Currently, EPCs are classified in two main sub-populations: early EPCs, also known as circulating angiogenic cells (CACs) and late EPCs or endothelial colony-forming cells (ECFCs). CACs have a hematopoietic like phenotype and they exert their regenerative activity through paracrine mechanisms while ECFCs have an endothelial phenotype and can differentiate into mature ECs, participating directly in blood vessels formation (Hur et al. 2004; Medina et al. 2017). SARS-CoV-2 infection could negatively affect the repairing properties of EPCs, interfering with the normal functioning of the cardiovascular system. However, not many studies have been done on how EPCs behave in COVID-19 patients.

A better understanding of the initial stages in which SARS-CoV-2 affects the endothelium, even in asymptomatic individuals, becomes crucial in order to predict or prevent unwanted secondary effects, and the risk of suffering from severe complications. In the current study, we have evaluated, by application of a mass spectrometry (MS)-based quantitative approach, the proteomic changes taking place in healthy CACs in response to the differential factors present in the serum of asymptomatic COVID-19 patients.

## Methods

### Study population

**Fig. 1:**
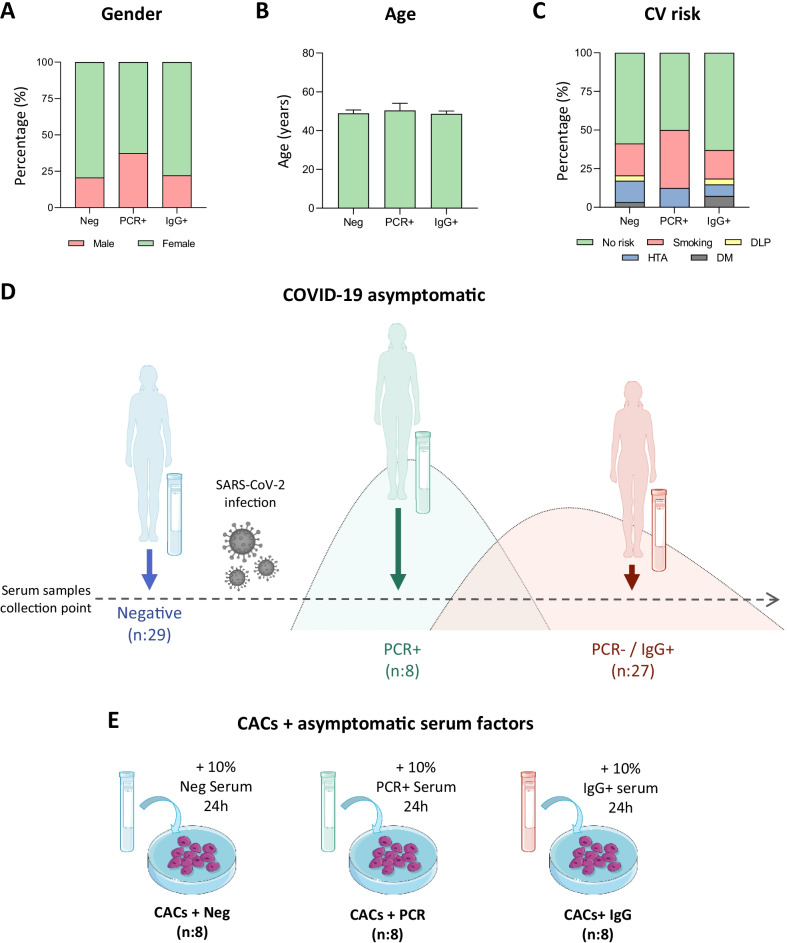
Study population characteristics and schematic representation of the experimental assay. A graphical representation of the donors’ characteristics is shown, including **A** Gender, **B** age and **C** Cardiovascular (CV) risks reported for each group. **D** Schematic representation of the infective stage of asymptomatic individuals at the time of serum extraction. Individuals were classified as COVID-19 negative (PCR −/IgG −, n:29), or COVID-19 positive, at the peak of infection (PCR + /IgG −, n:8) or after the infective peak (PCR −/IgG +, n:27). **E** CACs were incubated with the serum of COVID-19 negative donors, or with the serum of COVID-19 PCR + or COVID-19-IgG + asymptomatic patients

The study was conducted in asymptomatic donors recruited at the National Paraplegic Hospital (SESCAM), Toledo, Spain during April–May 2020. They were all workers of this hospital. A graphical representation of some characteristics registered for the study population is shown in Fig. 1A–C.

### Serum sample collection and tests performed for COVID-19 diagnostic

Briefly, peripheral blood samples were collected using serum separator tubes (SSTTM II advance, BD Vacutainer^®^), centrifuged (4000 g, 10 min, 4 °C) and stored at − 80 °C.

A SARS-CoV-2 qPCR analysis from nasopharyngeal samples was performed to determine the positive or negative status of the donors. Also, an ELISA assay testing for specific IgG and IgM antibodies (IME00136 and IME00137; Erba Mannheim) was performed with the serum previously collected. With all this information, donors were classified into three different groups: healthy donors with negative qPCR and antibody’s analysis test (Neg, n:29), asymptomatic patients with positive qPCR test for SARS-CoV-2 at blood extraction time (PCR + , n:8) and asymptomatic patients with positive IgG antibodies (IgG + , n:27) at the time of blood extraction (Fig. 1D).

### CACs isolation and culture

CACs were isolated from buffy coats from two healthy donors provided by the Andalusian Biobank Network (Decree 1/2013). Briefly, CACs were isolated from peripheral blood mononuclear cells (PBMCs) and cultured as previously described (Eslava-Alcon et al. 2020; Vega et al. 2017). PBMCs were isolated and plated in fibronectin coated plates (10 μg/ml) and incubated in EBM-2 media plus 10% fetal bovine serum (FBS) and Single Quots growth factors (Lonza). Non-adherent cells were discarded after four days and attached cells were allowed to grow in fresh media until day 7, when experimental assays were performed. CACs were characterized by flow cytometry assay, as described (Eslava-Alcon et al. 2020).

### CACs incubation ex vivo with patients’ serum

CACs (aprox. 1 million cells per group) were washed several times with PBS 1X, to discard any remaining traces of FBS from the initial conditioned media, and then incubated 24 h (37 °C, 10% CO_2_) with EBM-2 medium containing 10% serum of the Neg (CACs + Neg), PCR + /IgG − (CACs + PCR) or PCR −/IgG + groups (CACs + IgG), n:8 per group (Fig. 1E). After that, cells were collected using Trypsin–EDTA 1X (X0930-100; Biowest), centrifuged and washed once with PBS 1X, and snap frozen in liquid nitrogen before their storage at -80 ºC.

### Proteomic analysis

A label free quantitative (LFQ) MS approach was applied in order to identify differential protein levels between serum samples of asymptomatic donors (Neg n:29; PCR + n:8; IgG + n:27). Also, the protein changes in CACs after the incubation with the different sets of serum samples (CACs + Neg, n:8; CACs + PCR, n:8; CACs + IgG, n:8) were analyzed following the same LFQ approach.

Serum samples (10 μl) were supplemented with protease inhibitors (04693132001; Roche) and precipitated with acetone, over-night, centrifuged at 14,000 rpm, 25 min and the pellet resuspended in 8 M urea. Similarly, the cell pellets were resuspended in 50 μl of 8 M urea containing protease inhibitors (04693132001; Roche) for protein extraction and further proteomic analysis. For all samples, protein amount was quantified with the Qubit Fluorometric system (ThermoFisher Scientific) following manufacturer´s guidelines, and 50 µg of proteins in 8 M urea per sample were reduced (10 mM Dithiothreitol) and alkylated (50 mM Iodoacetamide). Samples were diluted four times with 50 mM ammonium bicarbonate and digested with Trypsin/LysC (V5073; Promega) (enzyme/substrate ratio 1:50) at 37 °C overnight. Finally, digestion was quenched with 0.1% TFA before peptide purification with C18 micro-columns, as described (Palmisano et al. 2010), and eluates were dried with a speed-vac system.

#### Liquid chromatography

A nanoElute high pressure nanoflow system (Bruker Daltonics) was connected to the timsTOF Pro, an ion-mobility quadrupole time of flight mass spectrometer (Bruker Daltonics) that uses the parallel accumulation-serial fragmentation (PASEF) acquisition method. Peptides were reconstituted in 0.1% formic acid (FA) up to a final concentration of 100 ng/μl and 200 ng were delivered to a Thermo Trap Cartridge (5 mm) column, and a reverse phase analytical column (25 cm × 75 um id IonOptics 25 cm, Thermo). Liquid chromatography was performed at 50ºC and peptides were separated on the analytical column using a 60 min gradient with buffers A (0.1% FA) and B (0.1% FA, Acetonitrile). For all samples, the TIMS-TOF Pro instrument was operated in data dependent acquisition (DDA) mode.

#### Data processing

Raw files were processed with MaxQuant (v 1.6.0.1), searching against a human protein database (Human UniProt) supplemented with contaminants. Carbamidomethylation of cysteines, oxidation of methionine and protein N-term acetylation were set as variable modifications. Minimal peptide length was set to 7 amino acids and a maximum of two tryptic missed-cleavages were allowed. Results were filtered at 1% FDR (peptide and protein level) and only proteins with at least two peptides identified were considered for further analysis. LFQ was done with match between runs (match window of 0.7 min and alignment window of 20 min). Afterwards, the “proteinGroup.txt” file was loaded in Perseus (v1.6.0.2) for further statistical analysis.

Proteins were considered as differentially expressed between groups when p-value < 0.05 and ratio > 1.5 (up-regulated) or ratio < 0.6 (down-regulated). Data processing was done using Venny v2.1 (Venn’s diagram), Perseus (hierarchical cluster), String (www.string-db.org), Enrichr (https://maayanlab.cloud/Enrichr), Ingenuity Pathway Analysis (IPA, Qiagen), Reactome (functional roles of proteins, www.reactome.org) and PINA v3 platform (protein interaction network analysis, www.omics.bjcancer.org/pina).

### Statistical analysis and machine learning

Protein quantification and statistics were obtained using MaxQuant (Tyanova et al. 2016a) and Perseus 1.6.15.0 (Tyanova et al. 2016b) software. Reverse database hits and contaminants were removed before performing a Student's T-test analysis with a multiple hypothesis correction of p-values (1% FDR). Differences were considered statistically significant when p-value < 0.05. Protein changes were confirmed with GraphPad Prism 9 software, and data were presented with box and plots graphs representing median, min and max value and showing all points. Also, receiver operating characteristic (ROC) curves were generated for differentially expressed proteins by plotting sensitivity (%) against 100%—specificity (%), indicating the area under the curve (AUC) and 95% confidence intervals.

In addition, we investigated the feasibility to perform two types of classification schemes based on protein levels using machine learning techniques: (a) a binary classification to discriminate between CACs + PCR *vs* CACs + Neg samples; and (b) a ternary classification into CACs treated with the serum from PCR + , IgG + asymptomatic and negative donors. Several supervised learning methods were applied in combination with a supervised attribute filter used to select features evaluating the worth of an attribute with a specified classifier (Deeb et al. 2015; Shi et al. 2021). Proteins were ranked according to their individual evaluations and the best 20 ranked ones were selected in each case.

Considering that complex models in small datasets limit generalization, low complexity models were used. In the case of the proposed ternary classification, performance metrics of linear support vector machines (SVM), Naïve Bayes (NB) and Random Forest algorithms were compared. For the binary classification, we compared linear SVM, NB, partial least squares discriminant analysis (PLS-DA), and least absolute shrinkage and selection operator (LASSO). In all cases, we combined the model-based prediction with feature selection to optimize the performance of the classifier and to identify strongly discriminative proteins. Accuracy was used as evaluation measure in the feature selection process. Both, the model training, and the feature selection, were done in a fivefold cross-validation procedure. The quality of classification was assessed using several parameters: accuracy, recall, true and false positive rate, and the area under the ROC curve. MATLAB (The MathWorks Inc., Natick, USA) and WEKA data mining software were used for building the models.

## Results

### Proteomic evaluation of asymptomatic COVID-19 patients’ serum

**Fig. 2:**
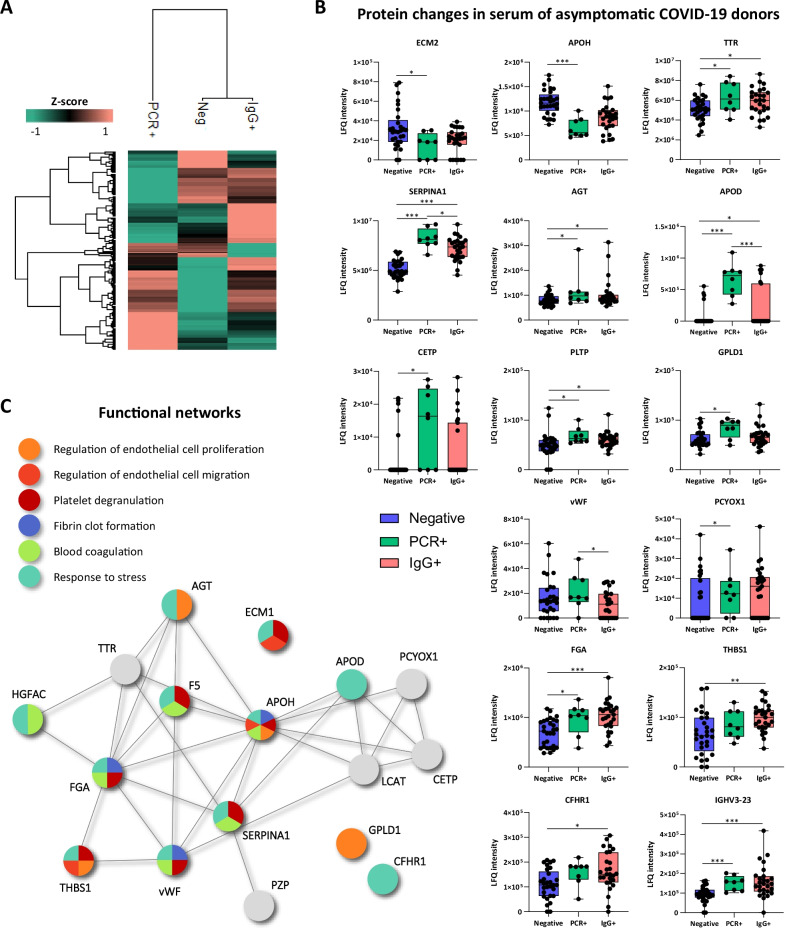
Proteins altered in asymptomatic patients’ serum and functional network. **A** Hierarchical clustering comparing the proteins patterns of the three groups analyzed. **B** Graphical representation of the label-free quantification (LFQ) intensities registered for proteins altered in the serum of COVID-19 PCR + (n:8) and COVID-19 IgG + asymptomatic patients (n:27) compared to COVID-19 negative donors (n:29). Differences were considered significant when p-values < 0.05. *p-value < 0.05, *p-value < 0.01, *p-value < 0.001. **C** Functional network, obtained with String on-line platform, highlighting the interactions detected between the serum proteins altered in the groups analyzed. Some of the most relevant functions identified for these proteins are represented

In total, 191 proteins were identified in serum by proteomic analysis (Additional file 1: Table S2). Among them, several proteins were altered in asymptomatic patients (PCR + /IgG − and PCR −/IgG + at the time of serum extraction), compared to COVID-19 negative subjects (Fig. 2). The differential protein patterns seen between groups are shown in a heat-map cluster (Fig. 2A). Proteins like TTR, SERPINA1, FGA, THBS1 or CFHR1 were up-regulated in the serum of PCR + and IgG + donors compared to negative individuals, showing significant differences between them (Fig. 2B). Others, like ECM1 or APOH, were down-regulated in PCR + and IgG + serums compared to negative controls. In some cases, like APOD or Cholesteryl ester transfer protein (CETP), the levels increased in PCR + donors while decreased (still higher than in controls) in the serum of IgG + individuals. These proteins participate, among others, in the coagulation cascade process, platelet degranulation (APOH, ECM1, SERPINA1), or regulation of endothelial cell migration (APOH, ECM1, THBS1) and proliferation (AGT, APOD, HGFAC, FGA, SERPINA1) (Fig. 2C). Also, according to IPA analysis, some of them have been associated with viral infection (APOD, APOH, SERPINA1), severe COVID-19 (APOD, APOH, PCYOX1), or leukocyte migration (APOD, IGHV3-13, IGHV3-23, SERPINA1, IGLC7, IGLV3-21).

### Molecular changes in CACs after incubation with COVID-19 serum samples

**Fig. 3:**
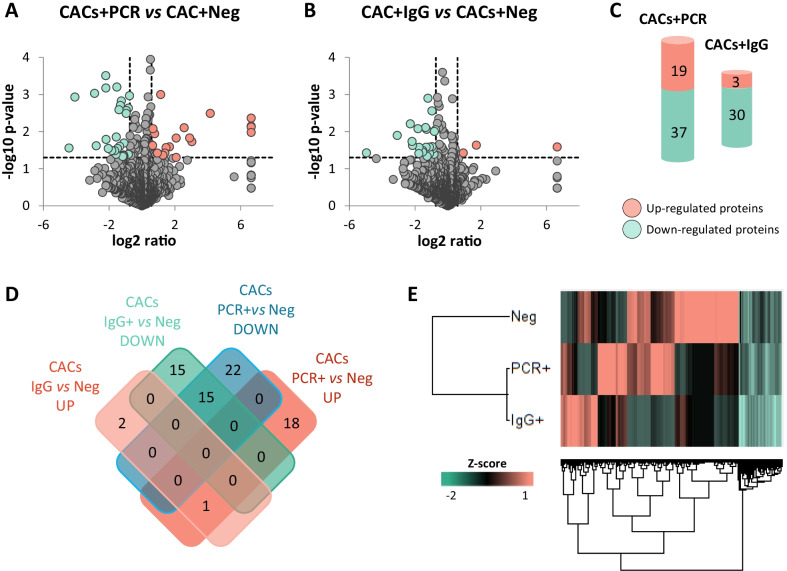
Proteomic changes in CACs in response to the serum of COVID-19 asymptomatic patients. Volcano plots representing proteins up- (red) or down- (green) regulated between CACs treated with **A** the serum of COVID-19 PCR + *vs* Negative donors (CACs + PCR), or **B** the serum of IgG + (CACs + IgG) *vs* COVID-19 negative donors (CACs + Neg). **C** Schematic representation of the number of proteins up- (red) or down- regulated (green) in CACs + PCR or CACs + IgG compared to CACs + Neg controls. **D** Venn’s diagram including the number of proteins up- or down-regulated, common or exclusive in CACs + PCR *vs* CACs + Neg, or in CACs + IgG *vs* CAC + Neg. **E** Hierarchical cluster representing the differential protein profiles for CACs + PCR, CACs + IgG or CACs + Neg

In total, 1438 proteins were identified in CACs incubated with the serum of COVID-19 negative (CACs + Neg), PCR + (CACs + PCR) and IgG + (CACs + IgG) asymptomatic donors (Additional file 1: Table S3). Furthermore, according to the LFQ analysis (Fig. 3A, B), several proteins were up-regulated in CACs + PCR (19 proteins) or CACs + IgG (3 proteins) compared to CACs + Neg controls (Fig. 3C). Also, other proteins were down-regulated (37 in CACs + PCR *vs* CACs + Neg and 30 in CACs + IgG *vs* CACs + Neg respectively) (Fig. 3C), while common alterations in both comparisons were identified too (Fig. 3D). A hierarchical classification of differentially expressed proteins indicated that the protein profiles of CACs in response to PCR + or IgG + serum were more similar between themselves than in CACs + Neg controls (Fig. 3E).

**Fig. 4:**
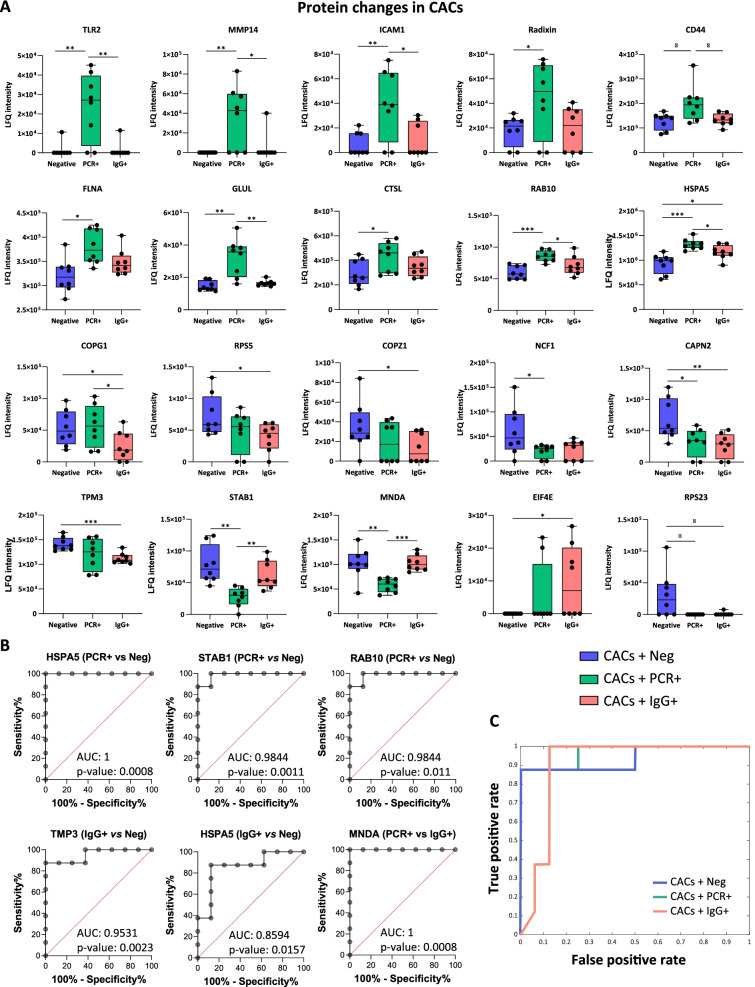
Proteins altered in CACs incubated with asymptomatic patients’ serum compared with negatives and functional network. **A** Graphical representation of the label-free quantification (LFQ) intensities registered for several proteins altered in CACs + PCR (n:8) and CACs + IgG (n:8) compared to CACs + Neg controls (n:8). Differences were considered significant when p-values < 0.05. *p-value < 0.05, *p-value < 0.01, *p-value < 0.001. **B** Receiver operating characteristic (ROC) analysis of HSPA5, STAB1, RAB10 and TMP3 proteins in asymptomatic COVID-19 patients with area under curve (AUC). **C** Naïve Bayes classifier

Proteins like Toll like receptor 2 (TLR2), Radixin, Matrix metalloproteinase 14 (MMP14), Intercellular adhesion molecule 1 (ICAM-1), CD44, GLUL, RAB10 or FLNA were significantly up-regulated in CACs + PCR, but the levels decreased in CACs + IgG. Similarly, proteins like Stabilin-1 (STAB1) or Myeloid cell nuclear differentiation antigen (MNDA), were down-regulated in the CACs + PCR group while recovered in CACs + IgG + serums. Other proteins (COPZ1, RPS23, CAPN2, NCF1) were down-regulated in both, CACs + PCR and CACs + IgG compared to CACs + Neg controls. The most relevant changes are shown in Fig. 4.

Some of these differentially expressed proteins were clearly discriminative for CACs in response to PCR + *vs* Negative serum or between CACs + IgG *vs* CACs + Neg groups, as indicated by the high AUCs values (Fig. 4B). Furthermore, several proteins stood out as result of applying machine learning algorithms (Additional file 1: Tables S4–6), including MNDA, STAB1, TLR2 or the Heat shock protein family A member 5 (HSPA5), among others. The built linear SVM, NB, PLS-DA, and LASSO models presented an accuracy of 1.00, achieving a maximum performance when classifying CACs + PCR and CACs + Neg treatments. Likewise, significant results were obtained with all these models (Table 1) when a ternary classification was applied to discriminate between CACs + PCR, CACs + IgG or CACs + Neg conditions. The NB classifier provided the best results, with an accuracy of 0.93 and a ROC area of 0.96 (Fig. 4C).

**Table 1:** Evaluation of different machine learning models to classify CACs samples incubated with serum of PCR +, IgG + and negative donors

	Accuracy	Recall	ROC area	Avg. TP rate	Avg. FP rate
Linear SVM	0.92	0.92	0.94	0.92	0.04
Naïve Bayes	0.93	0.92	0.96	0.92	0.04
Random forest	0.83	0.79	0.91	0.79	0.10

*ROC* receiver operating characteristic, *SVM* support vector machines, *TP* true positive, *FP* false positive

### Functional classification of proteins differentially expressed in CACs after incubation with COVID-19 serum samples

**Fig. 5:**
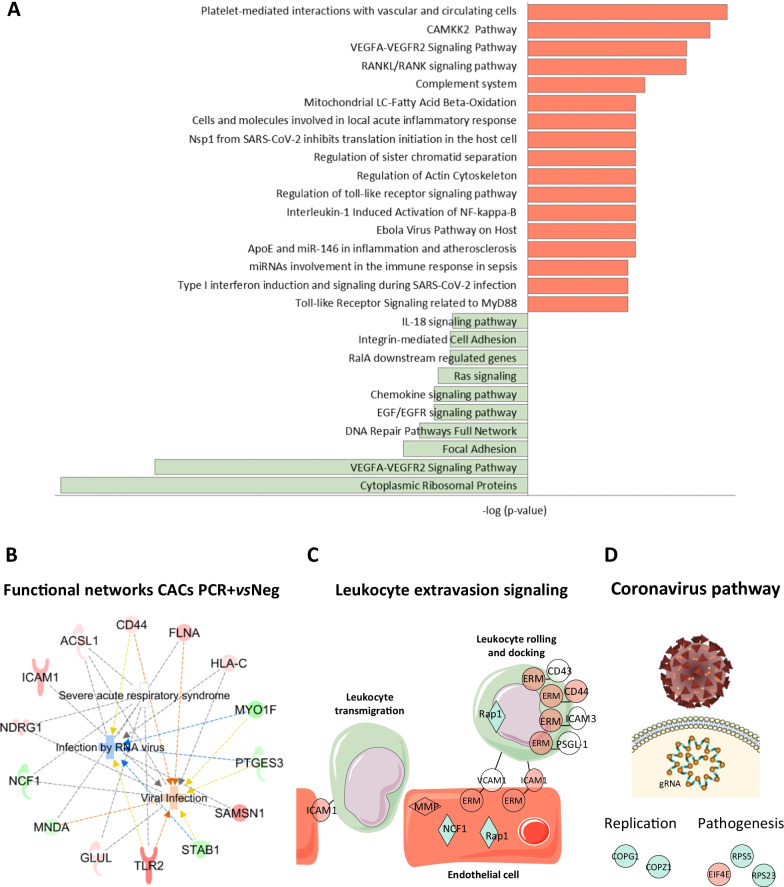
Functional classification of proteomic changes in CACs treated with the serum of PCR + *vs* Neg donors. **A** Altered pathways related with up- (red) and down- (green) regulated proteins in CACs + PCR *vs* CACs + Neg. **B** Ingenuity (IPA) functional network with proteins up- (red) or down-regulated (green) in CACs + PCR *vs* CACs + Neg, correlated with viral infection and severe acute respiratory syndrome (SARS), among others. **C** IPA graphical representation of proteins altered in CACs incubated with the serum of COVID-19 PCR + patients, compared to negative controls, participating in leukocyte extravasation signaling. **D** Proteins altered in CACs + IgG *vs* CACs + Neg related to viral pathogenesis and replication

The functional classification of differentially expressed proteins highlighted several major pathways altered in CACs + PCR (Fig. 5A). Moreover, according to IPA functional classification, several proteins altered in CACs in response to the PCR + serum have been previously linked to severe acute respiratory syndrome (SARS) or viral infection (Fig. 5B), together with leukocyte extravasation (Fig. 5C), among others. Similarly, some proteins altered in CACs + IgG were associated to coronavirus replication and its pathogenesis pathway (Fig. 4D). The most relevant functions of proteins altered in CACs + PCR cells are shown in Table 2.

**Table 2:** Functional classification of differentially expressed proteins in CACs after PCR + serum *vs* Neg serum incubation

Functions	P-value		Z-score	Molecules		Proteins
Cell movement	2.62E-04		0.602	CAPN2, CD44, FLNA, GLUL, ICAM1, MMP14, PRDX2, RALA, RAP1A, RDX, RTN4, SEPTIN9, SQSTM1, STAB1, TLR2		15
Cell Migration	3.07E-04		0.321	CAPN2, CD44, FLNA, GLUL, ICAM1, MMP14, PRDX2, RALA, RAP1A, RDX, RTN4, SEPTIN9, STAB1, TLR2		14
Viral infection	3.47E-04		0.401	ACSL1, CD44, FLNA, GLUL, HLA-C, ICAM1, MNDA, MYO1F, NCF1, NDRG1, PTGES3, SAMSN1, STAB1, TLR2		14
Apoptosis	1.78E-03		1.037	CD44, DYNLL1, EWSR1, FLNA, H1-0, MNDA, NDRG1, PPP2CA, PRDX2, RAP1A, RDX, RTN4, SQSTM1, TLR2		14
Necrosis	1.42E-02		0.284	CD44, DYNLL1, EWSR1, FLNA, ICAM1, IQGAP2, PPP2CA, PRDX2, RDX, RTN4, SQSTM1, TLR2		12
Cell survival	2.45E-04		0.943	CD44, EIF3A, FLNA, ICAM1, LILRB4, NDRG1, PRDX2, RPS11, SQSTM1, TLR2, XRCC5		11
Vasculogenesis	4.31E-06		1.545	CD44, FLNA, GLUL, ICAM1, MMP14, RAP1A, RDX, RTN4, STAB1, TLR2		10
Infection by RNA virus	1.88E-03		− 0.442	ACSL1, CD44, FLNA, HLA-C, MYO1F, PTGES3, SAMSN1, STAB1, TLR2		9
Cell activation	1.66E-04		0.669	CD44, EIF3A, FLNA, ICAM1, LILRB4, MMP14, PPP2CA, TLR2		8
Endothelial Cell Migration	4.28E-05		0.275	CD44, FLNA, GLUL, ICAM1, MMP14, RTN4, STAB1		7
Leukocytes Cell movement	1.09E-03	0.281			CD44, ICAM1, MMP14, RTN4, STAB1, TLR2	6
Phagocytes Migration	4.25E-05	0.453			CD44, ICAM1, MMP14, RTN4, STAB1	5
ROS production	2.71E-04	0.322			CD44, HVCN1, NCF1, PRDX2, TLR2	5

### Interaction networks between serum and CACs altered proteins

**Fig. 6:**
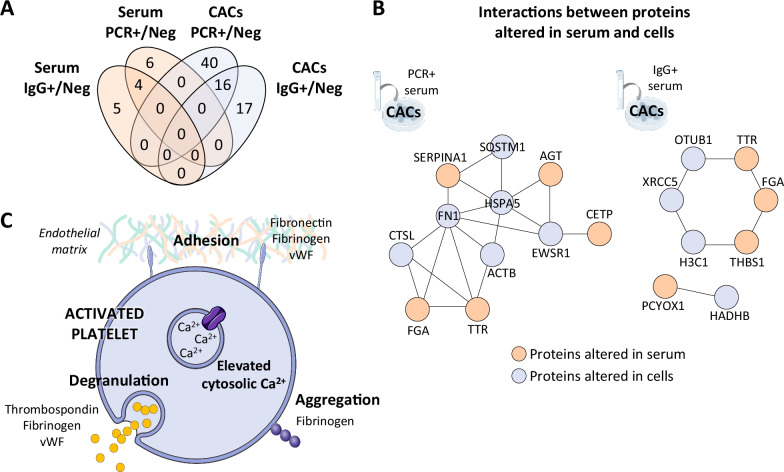
Interactions between proteins altered in serum and CACs samples. **A** Venn’s diagram including the number of proteins up- or down-regulated, common or exclusive in serum samples and CACs + PCR *vs* CACs + Neg and CACs + IgG *vs* CACs + Neg comparisons. **B** An in-silico analysis evaluating the potential interactions between altered proteins in the serum of COVID-19 asymptomatic donors (PCR + and IgG +) and the proteins altered in healthy CACs in response to those serums was performed with PINA v3 on-line platform. **C** One of the most representative functions found between the interactions found between both sets (serum and CACs) of altered proteins was platelet activation, including platelet aggregation and degranulation. Figure obtained with Reactome

An in-silico interaction network analysis was performed in order to find correlations between the proteins altered in the serum of COVID-19 asymptomatic donors and the protein changes in CACs in response to these factors (Fig. 6B). The networks found were mainly associated with platelet activation and signaling, as well as with extracellular matrix and activation of immune system in CACs. Several altered proteins in the serum of COVID-19 positive asymptomatic donors (FGA, SERPINA1, THBS1) and moreover, in CACs treated with these serum factors (HSPA5, FN1), have been associated with platelet aggregation and coagulation problems (Fig. 6C).

## Discussion

COVID-19 asymptomatic individuals or with mild symptoms present similar loads of SARS-CoV-2 virus in respiratory samples than symptomatic patients (Ra et al. 2021; You et al. 2021), representing a population that highly increases the risk of viral transmission due to the difficulties to identify and “isolate” them at the time of infection (Kronbichler et al. 2020; Gao et al. 2021). In addition, despite extraordinary research progress in the last two years, much is still unknown about the real impact of SARS-CoV-2 over the organism and the long-term consequences of such infection, even in asymptomatic individuals. In particular, the interaction of this virus with the cardiovascular system is still largely unknown.

To date, different studies have extensively analyzed the proteomic changes in serum, plasma or even urine from severe, critical, moderate or mild COVID-19 patients, in an attempt to identify potential signatures of the different stages of the disease (D’Alessandro et al. 2020; Messner et al. 2020; McArdle et al. 2021). However, not many studies have evaluated what happens in asymptomatic people. Herein, we have identified serum proteomic changes in asymptomatic individuals depending on the time of infection, including proteins up- or downregulated only at the highest infective peak (PCR + /IgG − in serum). These data corroborate that SARS-CoV-2 causes molecular alterations even in total or partial absence of classical symptoms. Many of the protein changes seen, mainly in PCR + serums, correlated to viral infection, platelet degranulation and leukocyte migration. These processes have already been described in severe COVID-19 patients (Shen et al. 2020; Shu et al. 2020).

Among them, CETP was up-regulated in the serum of asymptomatic individuals, as previously seen in COVID-19 patients with mild symptoms (Liu et al. 2021), while in the serum of critical patients this protein appeared down-regulated (Shu et al. 2020). CETP mediates lipid exchange (Satoh et al. 2016), but it also inhibits prolonged inflammation. Thus, CETP upregulation might correlate with the alteration of lipids after viral infection (membrane fusion, vesicles, etc.) (Abu-Farha et al. 2020), or even contribute to the lack of symptomatology in these patients (Shu et al. 2020). Similarly, plasma phospholipid transfer protein (PLTP) was up-regulated mainly in PCR + asymptomatic individuals. PLTP regulates lipoprotein metabolism, as well as inflammation and immune response, affecting Th1/Th2 polarization via modulation of IL18 expression (Desrumaux et al. 2016). Remarkably, these and other serum proteins related to lipid metabolism were previously seen in SARS-CoV patients, presenting an altered lipid and glucose metabolism even 12 years after infection (Wu et al. 2017). Further studies should evaluate the exact mechanisms by which coronaviruses affect lipid and glucose metabolism (Keihanian and Bigdelu 2020), since they could provide new insights regarding the adverse chronic cardiovascular complications.

Angiotensinogen (AGT) was also up-regulated in the serum of asymptomatic donors. AGT interacts with angiotensin converting enzyme 2 (ACE2), one of the main receptors responsible of SARS-CoV-2 entrance into the host cells which has been associated to COVID-19 cardiovascular complications (Wang et al. 2020; Wicik et al. 2020). In addition, other proteins up-regulated were thrombospondin (THBS1), fibrinogen α (FGA) or Von Willebrand factor (vWF), normally secreted by platelets during the degranulation process (Mehta and Yusuf 2003). These three proteins were already identified as procoagulant and thrombo-inflammatory markers in severe COVID-19 patients (Liu et al. 2021; Wool and Miller 2021; Zamanian-Azodi et al. 2021; Ward et al. 2021), but our data suggest that they are also altered at the peak of infection even in absence of symptoms. Since up-regulation of proteins like vWF correlates with inflammation, leaving the endothelium in a prothrombotic state (Ladikou et al. 2020; Escher et al. 2020), the potential long-term consequences of such endothelial damage in asymptomatic people should be tracked.

With this in mind, we next addressed whether the serum of asymptomatic COVID-19 individuals could affect basal endothelial cell function, by evaluating the protein changes taking place in CACs. Recent studies have reported an up-regulation of circulating EPCs levels even three months after SARS-CoV-2 infection, pointing them as vascular injury markers (Nizzoli et al. 2020). Thus, the effect of viral infection over these cells might help to explain potential cardiovascular secondary effects (Poyatos et al. 2021). Remarkably, many of the proteins altered in CACs incubated ex vivo with the serums of asymptomatic donors have been previously associated with viral infection, infection by RNA virus and SARS, but also to ECs movement or proliferation, and endothelial dysfunction. Besides, the alteration of proteins related to leukocyte extravasation and movement in CACs exposed to the serum of PCR + people, corroborates the activation of the immune process in these cells (Eslava-Alcon et al. 2020; Beltrán-Camacho et al. 2021; Medina et al. 2011). In response to pro-inflammatory stimuli such as viral infection, circulating EPCs initiate weak cell–cell interactions with the endothelium, promoting the expression of adhesion molecules such as E-selectin or ICAM-1 by these cells, which also promotes vascular permeability, EPCs adhesion and trans-endothelial migration (Krenning et al. 2009), as well as leukocyte recruitment (Othumpangat et al. 2016; Yu et al. 2020; Dai et al. 2008). Interestingly, ICAM-1 appeared up-regulated in CACs + PCR cells, but returned to basal levels in CACs + IgG. Elevated levels of ICAM-1 have been associated with severe endothelial dysfunction in severe COVID-19 patients (Nagashima et al. 2020; Tong et al. 2020). Also, the number of ICAM-1 + circulating EPCs appeared significantly increased in convalescent COVID-19 patients compared to healthy controls (Chioh et al. 2021). Thus, ICAM-1 levels might be indicative of the patient’s progression towards a worse condition or a prompt recovery without major consequences, at least at the short term. Similarly, MMP14 was only up-regulated in CACs + PCR cells. MMP14 promotes LDL receptors shedding (Alabi et al. 2021), and it also participates in tissue remodeling by degrading several extracellular matrix components (collagen, gelatin, fibronectin, etc.), which is usually associated to inflammation (Xia et al. 2021; Hwang et al. 2004). Overall, MMP14 may regulate the infiltration and migration of inflammatory precursor cells under arterial inflammation (Ries et al. 2007).

CD44, receptor for hyaluronic acid (HA) in adult ECs and CACs, was also up-regulated in CACs + PCR cells, in agreement with recent findings reporting high circulating HA levels in COVID-19 patients compared to healthy controls (Queisser et al. 2021). Similarly, heparin sulfate levels increased in HUVECs incubated with the plasma of COVID-19 patients, which was associated with endothelial glycocalyx shedding and degradation (Potje et al. 2021). Indeed, the glycocalyx becomes significantly damaged in severe COVID-19 patients, correlating with vascular damage in these patients (Queisser et al. 2021; Yamaoka-Tojo 2020; Teuwen et al. 2020). Finally, the incubation of human lung microvascular ECs with HA isolated from the plasma of COVID-19 patients, promoted endothelial barrier dysfunction in a CD44-depedent manner (Queisser et al. 2021).

Radixin was also over-expressed only in CACs + PCR. This protein shares 70% of the sequence with Ezrin, which appears to interact with the S spike protein of SARS-CoV, reducing viral entry (Millet et al. 2012), while in other cases like the human immunodeficiency virus-1 (HIV-1) Erzin enhances viral infectivity (Roy et al. 2014; Gadanec et al. 2021). Like Ezrin, Radixin might be exerting similar roles by modulating viral entry, although further studies should confirm such hypothesis.

Machine learning algorithms reported a list of proteins highly discriminating between the three groups compared (CACs + PCR, CACs + IgG or CACs + Neg). Among them, TLR2, up-regulated only in CACs + PCR, constitutes a cell surface innate immune sensor that can recognize several viral proteins upon infection (Oliveira-Nascimento et al. 2012), including the SARS-CoV-2 protein (Zheng et al. 2021). TLR2 activation in response to the SARS-CoV-2 E-protein promotes the production of pro-inflammatory cytokines such as TNF-α and INF-γ in vivo and in vitro*,* in both human and mice cells. Interestingly, the administration of TLR2 inhibitors to infected mice might protect against SARS-CoV-2 by impairing the release of cytokines (IL6, MCP1 or CXCL10) necessary for the development of the disease (Zheng et al. 2021; Sariol and Perlman 2021). Remarkably, TLR2 decreased significantly to basal levels in CACs + IgG. Thus, the blockade of TLR2 might prevent the progression of the disease towards a more severe stage. Different clinical trials using TLR-antagonists (M5049, MMG11, CuCpt22, hydroxychloroquine sulfate, imiquimod, etc.) are currently evaluating this therapeutic strategy (Gadanec et al. 2021; Patra et al. 2021; Grabowski et al. 2020).

The cell-surface receptor HSPA5, up-regulated mainly in CACs + PCR, has been proposed as an additional receptor for SARS-CoV-2 attachment and entry, together with ACE2, susceptible to viral recognition through the substrate-binding domain (Ha et al. 2020; Chu et al. 2018). Indeed, HSPA5 inhibitors interfere with SARS-CoV-2 infection (Palmeira et al. 2020), corroborating this hypothesis, while HSPA5 levels might predispose to a severe progression and outcome of COVID-19 in patients with older age, obesity, and diabetes (Shin et al. 2021).

MNDA was one of the most discriminating proteins highlighted by the predictive approaches. MNDA is required for INFα production from human blood cells in response to viruses (Gu et al. 2022). MNDA down-regulation in CAC + PCR might reflect down-regulation of INFα, a powerful antiviral factor, in an attempt of SARS-CoV-2 to endorse its own propagation and infectability (Gu et al. 2022). The application of IFNα therapy to COVID-19 patients resulted in accelerated viral clearance from the upper airways and in a reduction of the inflammatory biomarkers IL-6 and C-reactive protein (CRP) (Zhou et al. 2020). The fact that the MNDA went back to “normal” levels in CACs treated with IgG + serums could be indicative of cells overcoming the anti-viral blockade and cell post-infection recovery. Future studies should validate whether MNDA contributes indeed to the immune response to SARS-CoV-2.

Finally, the interactions detected between the altered serum factors and the protein changes in CACs correlated with platelet activation, degranulation and an activation of the coagulation cascade. Noteworthy, EPCs are known to modulate platelet’s function and they also seem to limit thrombogenic events by supporting vascular repair of injured areas (Li and Li 2016; Abou-Saleh et al. 2009). Given the few interactions found with this in silico approach, the changes in CACs might be promoted by additional serum proteins, not identified herein, or even by other molecules such as microRNA or exosomes present in serum after COVID-19 infection.

Several limitations of this study should be addressed, such as the fact that serum samples were collected at the early period of the pandemic, and the number of samples collected was limited. Furthermore, donors were recruited prior vaccination started, so the potential effect that vaccines could have over the endothelial response should be also evaluated in future assays. Similarly, CACs were obtained from two healthy donors from whom no information was provided due to data protection assignments. Future studies may determine whether the response seen in our study would be different depending on the “endothelial” donors’ profile (healthy vs individuals with certain pathologies).

## Conclusions

Overall, our results indicate that the ex-vivo incubation of CACs with the serum from COVID-19 asymptomatic patients promoted changes that resembled the effects associated to SARS-CoV-2 infection (inflammatory response, ECM disruption and vascular damage, among others). Remarkably, such processes are currently considered as the primary causes of COVID-19 related coagulopathy. Therefore, our model has proven to be effective to evaluate the effect of SARS-CoV-2 at the cellular level. The protein changes detected were different depending on the disease stage, when cells were exposed to serum of PCR + donors (at the highest peak of infection) or the serum of IgG + /PCR − patients that had already overcome the disease with no apparent symptoms. Some of the proteins identified here, such as TLR2, ICAM-1, CD44, HSPA5 or MNDA, might be considered as potential targets to inhibit the direct or indirect effects of SARS-CoV-2 on the endothelium and the vascular system. Further studies should evaluate whether the continuous alteration of these proteins correlates with the individual’s progression to a more severe condition or even with long-hauler sequelae or, on the contrary, their modulation could help to overcome the disease hopefully without major consequences.

## Supplementary Information

**Additional file 1: Table S1. **Serology test for antibodies detection results for PCR + samples. The table includes (from left to right): Number of serum sample, PCR test for virus detection results, ELISA test for IgM and IgG detection results. **Table S2.** Quantitative analysis of proteins differentially expressed in serum samples (*vs* Neg). The table includes (from left to right): Protein IDs (Uniprot accession number), protein description, PCR + /Neg ratio, PCR + /Neg p-value, IgG + /Neg ratio and IgG + /Neg p-value. Over-expressed values are indicated in red (considering up-regulated ratio > 1.5) and under-expressed values in green (down-regulated ratio < 0.6). The table shows the significant values for at least one of the comparisons (p-value < 0.05 as differentially significant). **Table S3.** Quantitative analysis of proteins differentially expressed in CACs incubated with serum samples of asymptomatic donors (vs Neg). The table includes (from left to right): Protein IDs (Uniprot accession number), protein description, PCR + /Neg ratio, PCR + /Neg p-value, IgG + /Neg ratio and IgG + /Neg p-value. Over-expressed values are indicated in red (considering up-regulated ratio > 1.5) and under-expressed values in green (down-regulated ratio < 0.6). The table shows the significant values for at least one of the comparisons (p-value < 0.05 as differentially significant). **Table S4.** Proteins highlighted by Naïve Bayes (NB) model for classifying CACs incubated with serum samples of asymptomatic donors (PCR + , IgG + and Negative). The analysis test mode used fivefold cross-validation. The table includes (from left to right): Protein IDs (Uniprot accession number), gen name and protein description. **Table S5.** Proteins highlighted by support vector machines (SVM) model for classifying CACs incubated with serum samples of asymptomatic donors (PCR + , IgG + and Negative). The analysis test mode used fivefold cross-validation. The table includes (from left to right): Protein IDs (Uniprot accession number), gen name and protein description. **Table S6.** Proteins highlighted by Random Forest model for classifying CACs incubated with serum samples of asymptomatic donors (PCR + , IgG + and Negative). The analysis test mode used fivefold cross-validation. The table includes (from left to right): Protein IDs (Uniprot accession number), gen name and protein description.

## Data Availability

All the data supporting the findings of this study have been provided within the article, together with online additional files. Also, proteomic results have been deposited to the ProteomeXchange Consortium via PRIDE partner repository (Perez-Riverol et al. 2019) (PXD030860).

## Abbreviations

ACE2: Angiotensin converting enzyme 2
AGT: Angiotensinogen
AUC: Area under the curve
CETP: Cholesteryl ester transfer protein
CACs: Circulating angiogenic cells
COVID-19: Coronavirus disease 2019
ECs: Endothelial cells
ECFCs: Endothelial colony-forming cells
EPCs: Endothelial progenitor cells
FBS: Fetal bovine serum
FGA: Fibrinogen α
HIV-1: Human immunodeficiency virus-1
HA: Hyaluronic acid
ICAM-1: Intercellular adhesion molecule-1
LFQ: Label free quantitative
LASSO: Least absolute shrinkage and selection operator
MS: Mass spectrometry
MMP14: Matrix metalloproteinase 14
NB: Naïve Bayes
PLS-DA: Partial least squares discriminant analysis
PBMCs: Peripheral blood mononuclear cells
PLTP: Plasma phospholipid transfer protein
ROC: Receiver operating characteristic
SARS-CoV-2: Severe acute respiratory syndrome coronavirus 2
SVM: Support vector machines
THBS1: Thrombospondin 1
TLR2: Toll like receptor 2
vWF: Von Willebrand factor
