# Supplementary material for: The serum of COVID-19 asymptomatic patients up-regulates proteins related to endothelial dysfunction and viral response in circulating angiogenic cells ex-vivo
Source: Mol Med. 2022 Apr 9;28:40. doi: 10.1186/s10020-022-00465-w (PMC8994070; doi:10.1186/s10020-022-00465-w)
Supplement: Supplementary file 1 — Additional file 1: Table S1. Serology test for antibodies detection results for PCR + samples. The table includes (from left to right): Number of serum sample, PCR test for virus detection results, ELISA test for IgM and IgG detection results. Table S2. Quantitative analysis of proteins differentially expressed in serum samples (vs Neg). The table includes (from left to right): Protein IDs (Uniprot accession number), protein description, PCR + /Neg ratio, PCR + /Neg p-value, IgG + /Neg ratio and IgG + /Neg p-value. Over-expressed values are indicated in red (considering up-regulated ratio > 1.5) and under-expressed values in green (down-regulated ratio < 0.6). The table shows the significant values for at least one of the comparisons (p-value < 0.05 as differentially significant). Table S3. Quantitative analysis of proteins differentially expressed in CACs incubated with serum samples of asymptomatic donors (vs Neg). The table includes (from left to right): Protein IDs (Uniprot accession number), protein description, PCR + /Neg ratio, PCR + /Neg p-value, IgG + /Neg ratio and IgG + /Neg p-value. Over-expressed values are indicated in red (considering up-regulated ratio > 1.5) and under-expressed values in green (down-regulated ratio < 0.6). The table shows the significant values for at least one of the comparisons (p-value < 0.05 as differentially significant). Table S4. Proteins highlighted by Naïve Bayes (NB) model for classifying CACs incubated with serum samples of asymptomatic donors (PCR + , IgG + and Negative). The analysis test mode used fivefold cross-validation. The table includes (from left to right): Protein IDs (Uniprot accession number), gen name and protein description. Table S5. Proteins highlighted by support vector machines (SVM) model for classifying CACs incubated with serum samples of asymptomatic donors (PCR + , IgG + and Negative). The analysis test mode used fivefold cross-validation. The table includes (from left to right): Protein IDs (Unipr [file 10020_2022_465_MOESM1_ESM.pdf]

Supplementary material

Endothelial progenitor cells protein changes in response to the serum factors of asymptomatic COVID-19 patients

Supplementary tables S1. Serology test for antibodies detection results for PCR+ samples. The table includes (from left to right): Number of serum sample, PCR test for virus detection results, ELISA test for IgM detection results and ELISA test for IgG detection results

Serum sample (nº)	PCR	IgM	IgG
066	+	-	-
081	+	-	-
090	+	-	-
111	+	-	-
163	+	-	+
169	+	-	-
195	+	-	-
299	+	-	-

Supplementary table S2. Quantitative analysis of proteins differentially expressed in serum samples (vs Neg). The table includes (from left to right): Protein IDs (Uniprot accession number), protein description, PCR+/Neg ratio, PCR+/Neg p-value, IgG+/Neg ratio and IgG+/Neg p-value. Over-expressed values are indicated in red (considering up-regulated ratio > 1.5) and under-expressed values in green (down-regulated ratio < 0.6). The table shows the significant values for at least one of the comparisons. (considering P-value < 0.05 as differentially significant)

Protein IDs	Protein names	Ratio PCR*vs*Neg	p-value	Ratio IgG*vs*Neg	p-value
A0A0G2JL54	C4a anaphylatoxin	1.728	0.002	1.545	0.001
C9JF17	Apolipoprotein D	10.883	0.000	3.232	0.030
P01743	Ig heavy chain V-I region HG3	3.351	0.008	3.172	0.001
P01764	Ig heavy chain V-III region 23	1.616	0.001	1.700	0.000
P20742	Pregnancy zone protein	4.099	0.091	3.049	0.018
P01766	Ig heavy chain V-III region BRO	3.289	0.008	1.918	0.063
P11597	Cholesteryl ester transfer protein	4.283	0.004	1.728	0.148
A0A0A0MS15	Immunoglobulin heavy variable 3-49	1.525	0.022	1.301	0.043
P01009	Alpha-1-antitrypsin	1.599	0.000	1.394	0.000
A0A0C4DH33	Immunoglobulin heavy variable 1-24	1.064	0.801	1.807	0.001
A0A0C4DH41	Ig heavy chain V-II region NEWM	1.149	0.727	1.590	0.025
A0A5H1ZRQ7	Ig lambda-7 chain C region	1.324	0.422	1.541	0.041
P02766	Transthyretin	1.238	0.020	1.159	0.009
P01019	Angiotensinogen	1.448	0.018	1.301	0.021
P01031	Complement C5	1.168	0.002	1.062	0.033
P02671	Fibrinogen alpha chain	1.396	0.020	1.490	0.000
P02763	Alpha-1-acid glycoprotein 1	1.261	0.023	1.182	0.009
P04217	Alpha-1B-glycoprotein	1.107	0.049	1.079	0.004
P22352	Glutathione peroxidase 3	1.052	0.519	1.104	0.029
A0A0C4DH31	Immunoglobulin heavy variable 1-18 precursor	1.083	0.496	1.193	0.011

A0A0G2JMB2	Immunoglobulin heavy constant alpha 2	0.946	0.776	1.297	0.037
B7ZKJ8	Inter-alpha-trypsin inhibitor heavy chain H4	0.959	0.436	1.066	0.043
Q9UK55	Protein Z-dependent protease inhibitor	1.263	0.124	1.248	0.008
P00450	Ceruloplasmin	1.078	0.446	1.135	0.014
P00740	Coagulation factor IX	0.944	0.737	1.150	0.044
P00742	Coagulation factor X	0.972	0.666	1.091	0.021
P01011	Alpha-1-antichymotrypsin	1.047	0.370	1.062	0.047
P01742	Ig heavy chain V-I region EU	0.953	0.776	1.314	0.047
P02750	Leucine-rich alpha-2-glycoprotein	1.111	0.350	1.183	0.013
P02753	Retinol-binding protein 4	1.193	0.111	1.150	0.023
P02765	Alpha-2-HS-glycoprotein	1.112	0.278	1.213	0.003
P02775	Platelet basic protein	0.949	0.602	1.103	0.046
P02776	Platelet factor 4	0.929	0.489	1.137	0.022
P02790	Hemopexin	1.064	0.138	1.098	0.001
P04196	Histidine-rich glycoprotein	1.135	0.198	1.134	0.015
P05543	Thyroxine-binding globulin	1.031	0.782	1.128	0.030
P05546	Heparin cofactor 2	0.997	0.969	1.153	0.004
P07996	Thrombospondin-1	1.289	0.238	1.476	0.001
P08185	Corticosteroid-binding globulin	1.111	0.173	1.108	0.014
P15169	Carboxypeptidase N catalytic chain	1.130	0.241	1.147	0.011
P18428	Lipopolysaccharide-binding protein	1.261	0.088	1.331	0.004
P19652	Alpha-1-acid glycoprotein 2	0.976	0.810	1.134	0.037
P22792	Carboxypeptidase N subunit 2	1.147	0.068	1.083	0.044
P25311	Zinc-alpha-2-glycoprotein	0.970	0.844	1.283	0.006
P55058	Phospholipid transfer protein	1.390	0.056	1.232	0.026
Q03591	Complement factor H-related protein 1	1.426	0.050	1.474	0.003
P12259	Coagulation factor V	1.419	0.049	1.149	0.117
P01857	Ig gamma-1 chain C region	0.689	0.002	0.872	0.985
P01859	Ig gamma-2 chain C region	0.604	0.001	0.861	0.967
E9PHK0	Tetranectin	0.858	0.031	0.944	0.936
O95445	Apolipoprotein M	0.813	0.027	0.974	0.687
P00748	Coagulation factor XII	0.791	0.007	0.828	1.000
P01834	Ig kappa chain C region	0.838	0.022	1.036	0.214
P02652	Apolipoprotein A-II	1.165	0.035	0.956	0.815
P04003	C4b-binding protein alpha chain	0.764	0.022	0.805	1.000
P06681	Complement C2	0.812	0.029	0.970	0.784
P08603	Complement factor H	0.872	0.049	0.860	1.000
P09871	Complement C1s subcomponent	0.881	0.016	0.983	0.702
P13671	Complement component C6	0.869	0.017	0.974	0.763
P80108	Phosphatidylinositol-glycan-specific phospholipase D	1.357	0.003	1.106	0.100
Q06033	Inter-alpha-trypsin inhibitor heavy chain H3	0.802	0.033	0.967	0.676
Q96PD5	N-acetylmuramoyl-L-alanine amidase	0.829	0.038	1.069	0.086
P80748	Ig lambda chain V-III region LOI	0.477	0.264	1.549	0.050
P02749	Beta-2-glycoprotein 1	0.554	0.000	0.732	1.000
Q16610	Extracellular matrix protein 1	0.453	0.029	0.624	0.995

Supplementary table S3. Quantitative analysis of proteins differentially expressed in CACs incubated with serum samples of asupmtomatic donors (vs Neg). The table includes (from left to right): Protein IDs (Uniprot accession number), protein description, PCR+/Neg ratio, PCR+/Neg p-value, IgG+/Neg ratio and IgG+/Neg p-value. Over-expressed values are indicated in red (considering up-regulated ratio > 1.5) and under-expressed values in green (down-regulated ratio < 0.6). The table shows the significant values for at least one of the comparisons. (considering P-value < 0.05 as differentially significant)

Protein IDs	Protein names	Ratio PCR*vs*Neg	p-value	Ratio IgG*vs*Neg	p-value
P08590	Myosin light chain 3	3.188	0.026	3.300	0.023
P06730	Eukaryotic translation initiation factor 4E	100	0.150	100	0.026
P50281	Matrix metalloproteinase-14	100	0.007	100	0.334
Q15042	Rab3 GTPase-activating protein catalytic subunit	100	0.004	100	0.334
A0A1W2PR11	HLA class I histocompatibility antigen, C alpha chain	1.916	0.038	1.714	0.100
Q14152	Eukaryotic translation initiation factor 3 subunit A	4.229	0.050	2.641	0.280
A0A0G2JQ10	Leukocyte immunoglobulin-like receptor subfamily B member 4	100	0.008	1.000	1.000
P16070	CD44 antigen	1.568	0.024	1.048	0.681
O43684	Mitotic checkpoint protein BUB3	4.245	0.015	1.273	0.801
O60603	Toll-like receptor 2	17.821	0.003	1.087	0.954
P05362	Intercellular adhesion molecule 1	5.924	0.008	1.491	0.590
P15104	Glutamine synthetase	2.207	0.001	1.121	0.179
P33121	Long-chain-fatty-acid--CoA ligase 1	1.708	0.011	1.094	0.078
P62857	40S ribosomal protein S28	1.596	0.008	1.104	0.592
Q13501	Sequestosome-1	2.762	0.027	1.085	0.876
Q92597	Protein NDRG1	2.636	0.038	1.102	0.808
Q9H939	Proline-serine-threonine phosphatase-interacting protein 2	100	0.011	1.000	1.000
P35241	Radixin	2.420	0.044	1.061	0.885
Q9NSI8	SAM domain-containing protein SAMSN-1	8.223	0.019	0.000	0.155
Q9C002	Normal mucosa of esophagus-specific gene 1 protein	7.460	0.015	0.000	0.149
E9PL57	NEDD8	1.363	0.383	1.909	0.038
P59998	Actin-related protein 2/3 complex subunit 4	0.655	0.000	0.739	0.000
P11021	78 kDa glucose-regulated protein	1.422	0.000	1.251	0.017
P14314	Glucosidase 2 subunit beta	1.296	0.001	1.200	0.036
O15127	Secretory carrier-associated membrane protein 2	1.278	0.003	1.264	0.035
P12236	ADP/ATP translocase 3	0.801	0.022	0.771	0.007
P13667	Protein disulfide-isomerase A4	1.304	0.004	1.204	0.039
P14866	Heterogeneous nuclear ribonucleoprotein L	0.694	0.008	0.758	0.025
P19338	Nucleolin	0.744	0.023	0.661	0.002
P20340	Ras-related protein Rab-6A	1.171	0.023	1.323	0.048
P23141	Liver carboxylesterase 1	1.225	0.018	1.169	0.047
P23246	Splicing factor, proline- and glutamine-rich	1.189	0.016	1.207	0.001
P23528	Cofilin-1	0.854	0.008	0.865	0.011
P49755	Transmembrane emp24 domain-containing protein 10	1.293	0.005	1.220	0.016
P62879	Guanine nucleotide-binding protein G(I)/G(S)/G(T) subunit beta-2	0.626	0.044	0.698	0.022
P63220	40S ribosomal protein S21	1.394	0.004	1.306	0.014
Q00325	Phosphate carrier protein, mitochondrial	0.673	0.020	0.669	0.049
Q15084	Protein disulfide-isomerase A6	1.241	0.010	1.223	0.024

Q99536	Synaptic vesicle membrane protein VAT-1 homolog	0.740	0.005	0.791	0.028
P13639	Elongation factor 2	0.930	0.371	0.782	0.009
P29966	Myristoylated alanine-rich C-kinase substrate	0.988	0.952	0.645	0.045
P62979	Ubiquitin-40S ribosomal protein S27a	0.896	0.339	0.676	0.005
P52272	Heterogeneous nuclear ribonucleoprotein M	0.796	0.068	0.683	0.008
P01903	HLA class II histocompatibility antigen, DR alpha chain	0.869	0.066	0.841	0.012
P04439	HLA class I histocompatibility antigen, A-3 alpha chain	1.357	0.062	1.266	0.044
P02545	Prelamin-A/C	1.111	0.074	1.158	0.013
J3KN67	Tropomyosin alpha-3 chain	0.853	0.108	0.793	0.000
O00182	Galectin-9	1.031	0.879	1.258	0.036
P04406	Glyceraldehyde-3-phosphate dehydrogenase	0.904	0.171	0.850	0.033
P28062	Proteasome subunit beta type-8	0.862	0.412	0.652	0.038
P60174	Triosephosphate isomerase	1.423	0.069	1.354	0.029
P62993	Growth factor receptor-bound protein 2	1.098	0.420	1.215	0.047
P63104	14-3-3 protein zeta/delta	0.937	0.086	0.872	0.000
P63241	Eukaryotic translation initiation factor 5A-1	0.862	0.250	0.691	0.002
P68363	Tubulin alpha-1B chain	0.888	0.069	0.843	0.011
P08865	40S ribosomal protein SA	1.274	0.044	0.992	0.923
P06454	Prothymosin alpha	1.164	0.031	0.986	0.823
O60763	General vesicular transport factor p115	1.391	0.023	1.068	0.549
P05387	60S acidic ribosomal protein P2	1.221	0.020	1.058	0.360
P13686	Tartrate-resistant acid phosphatase type 5	0.760	0.030	1.082	0.482
P13693	Translationally-controlled tumor protein	1.211	0.015	0.928	0.400
P21333	Filamin-A	1.186	0.004	1.086	0.098
P25398	40S ribosomal protein S12	1.179	0.031	1.025	0.732
P29350	Tyrosine-protein phosphatase non-receptor type 6	0.755	0.006	0.988	0.878
P51148	Ras-related protein Rab-5C	0.858	0.030	0.952	0.418
P61026	Ras-related protein Rab-10	1.430	0.000	1.181	0.118
Q9HCN8	Stromal cell-derived factor 2-like protein 1	1.457	0.002	1.026	0.878
P25786	Proteasome subunit alpha type-1	1.220	0.005	1.301	0.054
A0A494C0X7	Integrin beta-2	0.829	0.032	0.912	0.191
O75390	Citrate synthase, mitochondrial	0.766	0.004	0.911	0.368
O75347	Tubulin-specific chaperone A	1.309	0.003	1.079	0.543
E9PAV3	Nascent polypeptide-associated complex subunit alpha, muscle-specific form	1.229	0.042	1.040	0.605
O15143	Actin-related protein 2/3 complex subunit 1B	1.123	0.018	1.089	0.145
P06744	Glucose-6-phosphate isomerase	1.233	0.007	1.139	0.095
P07602	Prosaposin	0.628	0.031	0.856	0.283
P07711	Cathepsin L1	1.442	0.036	1.169	0.302
P09496	Clathrin light chain A	1.131	0.049	0.993	0.931
P11940	Polyadenylate-binding protein 1	1.351	0.027	1.140	0.075
P12956	X-ray repair cross-complementing protein 6	0.607	0.024	0.797	0.292
P14625	Endoplasmin	1.173	0.029	1.083	0.328
P17050	Alpha-N-acetylgalactosaminidase	0.782	0.023	0.869	0.094
P23526	Adenosylhomocysteinase	1.161	0.049	1.100	0.215
P27105	Erythrocyte band 7 integral membrane protein	1.380	0.029	1.165	0.250
P30040	Endoplasmic reticulum resident protein 29	1.336	0.009	1.171	0.126

P30101	Protein disulfide-isomerase A3	1.153	0.006	1.110	0.138
P42785	Lysosomal Pro-X carboxypeptidase	0.717	0.042	0.743	0.121
P46940	Ras GTPase-activating-like protein IQGAP1	0.605	0.013	0.805	0.277
P49748	Very long-chain specific acyl-CoA dehydrogenase, mitochondrial	1.373	0.040	1.170	0.445
P53634	Dipeptidyl peptidase 1	0.810	0.016	0.872	0.075
P60709	Actin, cytoplasmic 1	1.418	0.021	1.241	0.203
P61586	Transforming protein RhoA	1.161	0.018	1.077	0.290
P67936	Tropomyosin alpha-4 chain	1.334	0.020	1.194	0.061
Q16719	Kynureninase	1.256	0.031	1.086	0.137
Q5Y7D1	HLA class II histocompatibility antigen DR beta chain	0.874	0.028	0.959	0.604
Q7KZF4	Staphylococcal nuclease domain-containing protein 1	0.684	0.045	0.878	0.374
Q9H299	SH3 domain-binding glutamic acid-rich-like protein 3	1.110	0.022	1.014	0.850
Q9UJU6	Drebrin-like protein	1.109	0.034	1.061	0.230
Q9Y6N5	Sulfide:quinone oxidoreductase, mitochondrial	1.351	0.033	1.150	0.163
Q9Y678	Coatomer subunit gamma-1	1.061	0.831	0.460	0.039
Q96FW1	Ubiquitin thioesterase OTUB1	0.625	0.102	0.512	0.025
P46782	40S ribosomal protein S5	0.657	0.146	0.535	0.029
P11908	Ribose-phosphate pyrophosphokinase 2	0.841	0.655	0.293	0.039
P21291	Cysteine and glycine-rich protein 1	0.784	0.209	0.591	0.009
P84077	ADP-ribosylation factor 1	0.745	0.140	0.562	0.010
Q09028	Histone-binding protein RBBP4	0.807	0.634	0.220	0.019
Q13200	26S proteasome non-ATPase regulatory subunit 2	0.739	0.283	0.556	0.041
P19105	Myosin regulatory light chain 12A	0.591	0.001	0.643	0.001
Q9NQC3	Reticulon-4	0.420	0.002	0.638	0.006
P62917	60S ribosomal protein L8	0.000	0.032	1.189	0.779
P07305	Histone H1.0	0.241	0.029	0.939	0.872
P41218	Myeloid cell nuclear differentiation antigen	0.565	0.002	1.002	0.986
P63167	Dynein light chain 1, cytoplasmic	0.564	0.041	1.042	0.780
Q13576	Ras GTPase-activating-like protein IQGAP2	0.444	0.047	0.734	0.322
Q86V81	THO complex subunit 4	0.317	0.033	1.073	0.854
Q9NY15	Stabilin-1	0.347	0.001	0.800	0.250
Q9Y2S2	Lambda-crystallin homolog	0.218	0.000	0.931	0.697
Q15185	Prostaglandin E synthase 3	0.463	0.043	0.914	0.693
Q01844	RNA-binding protein EWS	0.459	0.044	0.696	0.265
O00160	Unconventional myosin-If	0.280	0.033	0.659	0.313
P56537	Eukaryotic translation initiation factor 6	0.000	0.022	0.682	0.599
P62244	40S ribosomal protein S15a	0.380	0.003	0.729	0.247
P62834	Ras-related protein Rap-1A	0.517	0.003	0.677	0.073
P38571	Lysosomal acid lipase/cholesteryl ester hydrolase	0.220	0.016	0.344	0.036
P62266	40S ribosomal protein S23	0.000	0.032	0.032	0.038
P62308	Small nuclear ribonucleoprotein G	0.000	0.006	0.000	0.006
P11233	Ras-related protein Ral-A	0.046	0.028	0.000	0.021
Q96EP5	DAZ-associated protein 1	0.392	0.025	0.283	0.008
O14880	Microsomal glutathione S-transferase 3	0.059	0.001	0.204	0.006
O43598	2-deoxynucleoside 5-phosphate N-hydrolase 1	0.000	0.008	0.000	0.008
P14854	Cytochrome c oxidase subunit 6B1	0.218	0.001	0.405	0.009

P17655	Calpain-2 catalytic subunit	0.468	0.020	0.389	0.008
P32969	60S ribosomal protein L9	0.032	0.000	0.032	0.000
P55084	Trifunctional enzyme subunit beta, mitochondrial	0.403	0.002	0.509	0.003
P67775	Serine/threonine-protein phosphatase 2A catalytic subunit alpha isoform	0.357	0.029	0.344	0.032
P68431	Histone H3.1	0.000	0.049	0.000	0.049
Q6FI13	Histone H2A type 2-A	0.000	0.005	0.118	0.013
Q9UHD8	Septin-9	0.515	0.003	0.429	0.001
P13010	X-ray repair cross-complementing protein 5	0.489	0.057	0.399	0.027
Q96D96	Voltage-gated hydrogen channel 1	0.442	0.059	0.415	0.046
P46063	ATP-dependent DNA helicase Q1	0.371	0.063	0.295	0.027
Q08211	ATP-dependent RNA helicase A	0.485	0.139	0.224	0.024
Q86U42	Polyadenylate-binding protein 2	0.456	0.287	0.000	0.022
Q9HB90	Ras-related GTP-binding protein C	0.395	0.238	0.000	0.030
Q9Y6W5	Wiskott-Aldrich syndrome protein family member 2	0.558	0.096	0.459	0.026
O95881	Thioredoxin domain-containing protein 12	0.471	0.047	0.481	0.056
P02751	Fibronectin	0.000	0.033	0.543	0.421
P05386	60S acidic ribosomal protein P1	0.339	0.014	0.550	0.118
Q71UI9	Histone H2A.V	0.136	0.001	0.506	0.075
P14598	Neutrophil cytosol factor 1	0.335	0.041	0.385	0.066
P32119	Peroxiredoxin-2	0.145	0.024	0.274	0.081

Supplementary table S4. Proteins highlighted by Naïve Bayes (NB) model for classifying CACs incubated with serum samples of asymptomatic donors (PCR+, IgG+ and Negative). The analysis test mode used 5-fold cross-validation. The table includes (from left to right): Protein IDs (Uniprot accession number), gen name and protein description.

Protein IDs	Gen name	Name
P17050	NAGA	Alpha-N-acetylgalactosaminidase
P15104	GLUL	Glutamine synthetase
O14880	MGST3	Microsomal glutathione S-transferase 3
P41218	MNDA	Myeloid cell nuclear differentiation antigen
P25786	PSMA1	Proteasome subunit alpha type-1
P59998	ARPC4	Actin-related protein 2/3 complex subunit 4
P55084	HADHB	Trifunctional enzyme subunit beta, mitochondrial
P11021	HSPA5	78 kDa glucose-regulated protein
P33121	ACSL1	Long-chain-fatty-acid--CoA ligase 1
J3KN67	TPM3	Tropomyosin alpha-3 chain
P09496	CLTA	Clathrin light chain A
P38571	LIPA	Lysosomal acid lipase/cholesteryl ester hydrolase
P62857	RPS28	40S ribosomal protein S28
Q9NQC3	RTN4	Reticulon-4
P13639	EEF2	Elongation factor 2
Q9NSI8	SAMSN1	SAM domain-containing protein SAMSN-1
O15127	SCAMP2	Secretory carrier-associated membrane protein 2
P19105	MYL12A	Myosin regulatory light chain 12A
Q9Y6N5	SQOR	Sulfide:quinone oxidoreductase, mitochondrial
Q9C002	NMES1	Normal mucosa of esophagus-specific gene 1 protein

Supplementary table S5. Proteins highlighted by support vector machines (SVM) model for classifying CACs incubated with serum samples of asymptomatic donors (PCR+, IgG+ and Negative). The analysis test mode used 5-fold cross-validation. The table includes (from left to right): Protein IDs (Uniprot accession number), gen name and protein description.

Protein IDs	Gen name	Name
Q71UI9	H2AZ2	Histone H2A.V
P32969	RPL9	60S ribosomal protein L9
Q9Y277	VDAC3	Voltage-dependent anion-selective channel protein 3
P14854	COX6B1	Cytochrome c oxidase subunit 6B1
O14672	ADAM10	Disintegrin and metalloproteinase domain-containing protein 10
P11233	RALA	Ras-related protein Ral-A
P62308	SNRPG	Small nuclear ribonucleoprotein G
O14880	MGST3	Microsomal glutathione S-transferase 3
Q9Y2S2	CRYL1	Lambda-crystallin homolog
P21980	TGM2	Protein-glutamine gamma-glutamyltransferase 2
Q6FI13	H2AC18/9	Histone H2A type 2-A
A0A0G2JQ10	LILRB4	Leukocyte immunoglobulin-like receptor subfamily B member 4
Q9UBS4	DNAJB11	DnaJ homolog subfamily B member 11 precursor
Q9H939	PSTPIP2	Proline-serine-threonine phosphatase-interacting protein 2
Q9NPJ3	ACOT13	Acyl-coenzyme A thioesterase 13
O60603	TLR2	Toll-like receptor 2
P62917	RPL8	60S ribosomal protein L8
Q6P4A8	PLBD1	Phospholipase B-like 1 precursor
Q9BZZ5	API5	Apoptosis inhibitor 5
P50281	MMP14	Matrix metalloproteinase-14

Supplementary table S6. Proteins highlighted by Random Forest model for classifying CACs incubated with serum samples of asymptomatic donors (PCR+, IgG+ and Negative). The analysis test mode used 5-fold cross-validation. The table includes (from left to right): Protein IDs (Uniprot accession number), gen name and protein description.

Protein IDs	Gen name	Name
P62424	RPL7A	60S ribosomal protein L7a
P17050	NAGA	Alpha-N-acetylgalactosaminidase
P54727	RAD23B	UV excision repair protein RAD23 homolog B
P13693	TPT1	Translationally-controlled tumor protein
P08631	HCK	Tyrosine-protein kinase HCK
P41218	MNDA	Myeloid cell nuclear differentiation antigen
Q9NY15	STAB1	Stabilin-1
P84077	ARF1	ADP-ribosylation factor 1
P61586	RHOA	Transforming protein RhoA
Q15008	PSMD6	26S proteasome non-ATPase regulatory subunit 6
Q9H299	SH3BGRL3	SH3 domain-binding glutamic acid-rich-like protein 3
P11279	LAMP1	Lysosome-associated membrane glycoprotein 1
P12236	SLC25A6	ADP/ATP translocase 3
O75369	FLNB	Filamin-B
Q92597	NDRG1	Protein NDRG1

P41567	EIF1	
P18621	RPL17	
P53634	CTSC	Dipeptidyl peptidase 1
P14854	COX6B1	Cytochrome c oxidase subunit 6B1
P40939	HADHA	
